# Congenital aniridia with cataract: case series

**DOI:** 10.1186/s12886-017-0503-6

**Published:** 2017-07-04

**Authors:** Jin Da Wang, Jing Shang Zhang, Ying Xiong, Jing Li, Xiao Xia Li, Xue Liu, Jing Zhao, Frank F. Tsai, Jhanji Vishal, Qi Sheng You, Yao Huang, Xiu Hua Wan

**Affiliations:** 1 Beijing Institute of Ophthalmology, Beijing Tongren Eye Center, Beijing Tongren Hospital of Capital Medical University, Beijing Key Laboratory of Ophthalmology and Visual Sciences, Beijing, 100005 China; 2 Beijing Tongren Eye Center, Beijing Tongren Hospital of Capital Medical University, Beijing Key Laboratory of Ophthalmology and Visual Sciences, Beijing, 100005 China; 3 Jacobs Retina Center, Shiley Eye Institute, and Department of Ophthalmology, University of California, La Jolla, San Diego, California USA; 4 Department of Ophthalmology and Visual Sciences, the Chinese University of Hong Kong, Hong Kong, China

**Keywords:** Congenital aniridia, Cataract removal surgery, Color artificial lens, Capsular tension ring

## Abstract

**Background:**

This study evaluates patients with congenital aniridia and cataract who underwent phacoemulsification, capsular tension ring placement, and foldable intraocular lens implantation.

**Methods:**

In this prospective case series, 10 patients (17 eyes) underwent cataract surgery via a 3.2 mm clear corneal incision. A continuous circular capsulorhexis with <6 mm diameter was employed. A capsular tension ring and HOYA yellow foldable posterior chamber intraocular lens was implanted. All patients wore color contact lenses postoperatively. Paired t test was used to compare visual acuity, intraocular pressure, and corneal endothelial changes before and after surgery.

**Results:**

A single surgeon performed all surgeries. The best-corrected visual acuity improved from value 1.03 ± 0.27LogMAR preoperatively to value 0.78 ± 0.26LogMAR postoperatively (*p* = 0.000). The photophobic symptoms improved significantly after surgery. The mean corneal endothelial cell density before and after surgery was 3280 ± 473 cells/mm2 and 2669 ± 850 cells/mm2, respectively (*p* = 0.006). None of the patients developed corneal endothelial decompensation or secondary glaucoma after surgery.

**Conclusions:**

Treatment of congenital aniridia and coexistent cataract by phacoemulsification, posterior chamber foldable lens implantation, capsular tension ring placement was safe and effective. Use of colored contact lenses in the postoperative period can reduce photophobic symptoms in this group of patients.

**Trial registration:**

ChiCTR-OOC-17011638 (retrospectively registered at 12,June,2017)

## Background

Congenital aniridia is a rare genetic eye disease due to PAX6 mutation. It is associated with neuroectodermal and mesodermal dysplasia [1, 2]. Congenital aniridia is often associated with cataract that may require surgery [1–5]. These cataracts are characterized by an early age of onset, higher risk of complications, and limited postoperative visual improvement [6–10].

Implantation of prosthetic iris devices during cataract surgery has been described to alleviate the photophobia symptoms induced by aniridia, although serious complications such as secondary glaucoma and corneal endothelial decompensation may occur after surgery [6, 11].

In this study we describe the surgical and visual outcomes of cataract surgery in cases with congenital aniridia using phacoemulsification, posterior chamber foldable lens implantation, and capsular tension ring (CTR) placement.

## Methods

### Subjects

The study complied with the tenets of the Declaration of Helsinki and was approved by the Ethics Board of the Beijing Tongren Hospital. Written, informed consent was obtained from all patients before surgery. A total of 10 patients (17 eyes) with congenital aniridia and cataract were operated between January 2011 and December 2014. Patients with serious keratopathy (effect the patients’ visual acuity), glaucoma or lens dislocation were excluded.

### Examinations

All patients underwent preoperative slit lamp examination of the anterior segment and intraocular pressure (IOP) measurement. Fundus photography and macular optical coherence tomography (OCT) were performed. Optometry examination was performed to determine the refraction and best-corrected visual acuity (BCVA). Corneal endothelium cell density was measured using endothelial microscopy. The corneal curvature was measured with computerized keratometry and axial length was measured with A scan ultrasound. The SRK-T formula was used to calculate intraocular lens (IOL) power. Postoperative follow-up was performed at 1 week, then at 1, 3, 6, 12, and 24 months.

### Surgery

A single surgeon (WX) performed standard phacoemulsification and IOL implantation in all cases. All patients received tropicamide eye drops 1 h before the surgery. Topical anesthesia was applied 3 times within the conjunctival fornix 5–10 min before surgery. A 3.2-mm clear corneal wound was created with a keratome. Continuous circular capsulorhexis (CCC) was performed making sure that the largest diameter of the CCC was less than 6 mm. After phacoemulsification and irrigation-aspiration of the residual cortical matter, a capsular tension ring was implanted in the capsular bag through the 3.2 mm incision. Subsequently, a blue light absorptive yellow HOYA (manufacturer details needed, Japan) posterior chamber foldable IOL was implanted in the capsular bag. Postoperative treatment was started in the form of antibiotic eye drops (levofloxacin) and corticosteroid eye drops (prednisolone acetate) for 6 weeks. Patients were instructed to wear colored contact lenses 6–8 weeks after the surgery.

Statistical analysisStatistical analysis was performed using SPSS for Windows (version 22.0; IBM-SPSS, Chicago, IL, USA). Paired *t*-test was used to compare preoperative and postoperative BCVA, IOP, and corneal endothelial cell density. *P*-values represent results for 2-sided tests, with values less than 0.05 considered statistically significant.

## Results

**Fig. 1:**
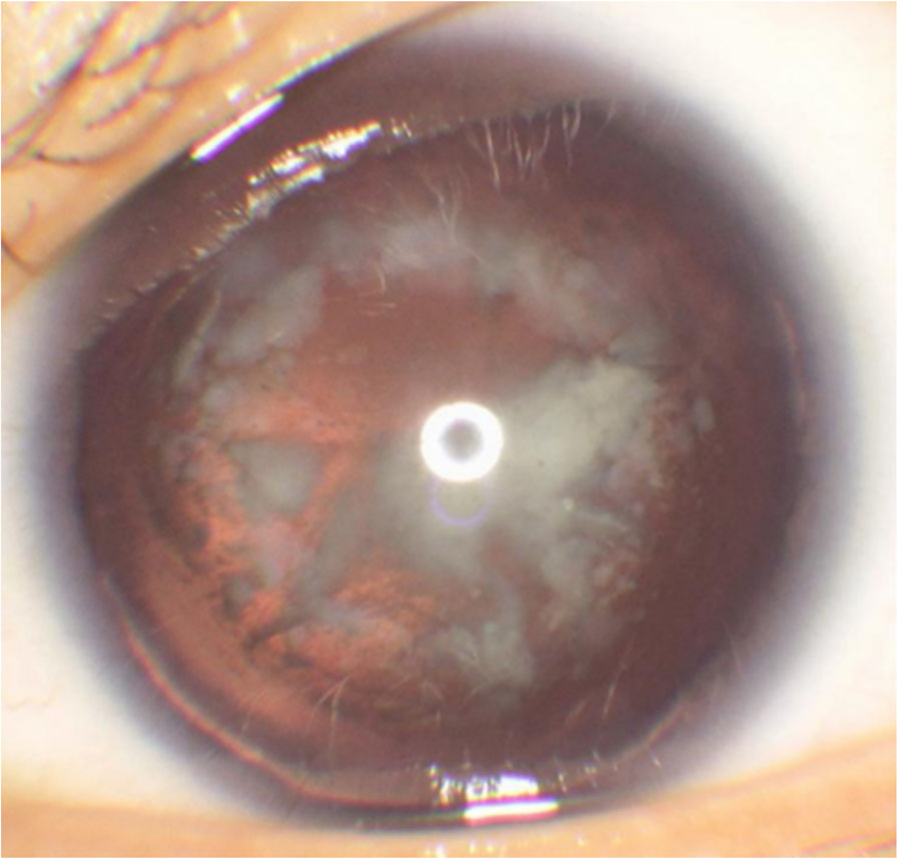
The lens opacity of the congenital aniridia complicated with cataract patient before surgery

A total of 17 eyes of 10 patients (6 males and 4 females) with congenital aniridia and cataract were included in this study (Tables 1 and 2). The mean age of the patients was 25.4 ± 14.77 years (range: 4 to 50 years). The mean preoperative LogMAR BCVA was 1.03 ± 0.27 (range: from 0.7 to 1.3) and the mean IOP was 16.35 ± 3.9 mmHg (range: 8-24 mmHg). The mean corneal endothelial cell density was 3280 ± 473 cells/mm^2^ (range: 1825 ~ 3829 cells/mm^2^) and mean axial length was 22.83 ± 1.98 mm (range: 20.44∼26.14 mm). Only 1 eye had a portion of iris present, while all of the other cases only had an iris root. All patients’ lenses were fully exposed with visible lens zonules at the time of the surgery (Fig. 1). All patients suffered from severe photophobia symptoms and varying degrees of nystagmus. Although most fundus photos appeared fairly normal, OCT images showed an absent or abnormal foveal morphology.

**Table 1:** Demographic and clinical information

Characteristics	Results
Gender	
Male	6
Female	4
Age at surgery	
Range	4 to 50 years
Mean	25.4 ± 14.77 years
Family history	
Yes	8 patients
No	2 patients
Laterality	
Bilateral	17 eyes
Unilateral	0
Extent of aniridia	
Total eyes	16 eyes
Partial eyes	1 eyes
Follow-up period after surgery	
1 month	1 eye
6 months	9 eyes
18 months	6 eyes
Lost	1 eye

**Table 2:** Clinical manifestations

Clinical features	Results
Lens opacity type	
Nuclear	5 eyes
Posterior capsule	4 eyes
Cortex	8 eyes
Cornea	
Mild surounding opacity	3 eyes
Normal	14 eyes
Foveal hypoplasia	17 eyes
Glaucoma	0
Nystagmus	17 eyes
IOP	
Range	8 to 24 mmHg
Mean	16.35 ± 3.9 mmHg
Central corneal thickness	
Range	530 to 688 um
Mean	612 ± 37 um
Endothelial cell count	
Range	1825 to 3829 cells/mm2
Mean	3280 ± 473 cells/mm^2^
Axial length	
Range	20.44 to 26.14 mm
Mean	22.83 ± 1.98 mm
IOL diopter	
Range	19.5 to 30 D
Mean	24.44 ± 4.3 D

All surgeries were performed successfully completed without any intraoperative complications. The power of implanted IOL ranged between 19.5 and 30.00D with an average of 24.44 ± 4.30 D.

**Fig. 2:**
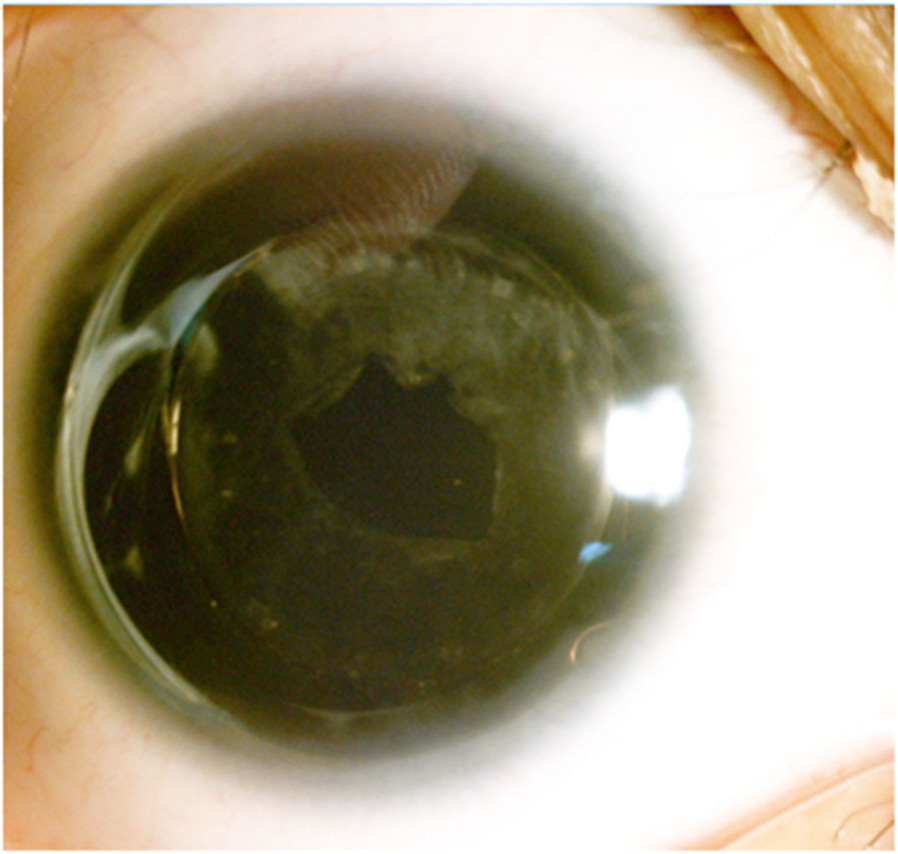
The posterior capsule opacity were treated by YAG laser at 6 months after cataract surgery

All patients followed up for a mean period of 10.2 ± 6.4 months postoperatively. One patient (1 eye) was lost to follow-up. One eye was followed up for 1 month after the surgery, 9 eyes were followed up for 6 months after the surgery, and 6 eyes were followed up for 18 months after the surgery. The mean postoperative LogMAR BCVA was 0.78 ± 0.26 (range: 0.5 to 1.3), (*p* = 0.000). The photphobic symptoms improved subjectively after surgery that was more obvious after wearing cosmetic contact lenses. All corneas remained clear at last follow up. There were no complications from wearing the colored contact lenses. The capsule bags of 4 eyes appeared fibrosed 6 months after the surgery. YAG laser treatment was performed for posterior capsule opacification (PCO) in 2 adult patients (4 eyes) 6 months after surgery (Fig. 2). There were no cases of IOL dislocation due to zonular weakness. No secondary glaucoma was observed. The mean corneal endothelial cell density decreased from 3280 ± 473 cell/mm^2^ *to* 2669 ± 850 cells/mm^2^ (*p* = 0.006), but no case with secondary corneal endothelial decompensation was observed.

## Discussion

The currently reported incidence of cataract in patients with congenital aniridia is approximately 50–85% [12]. Most researchers believe congenital aniridia complicated with cataract does not need to be treated when lens opacity and its effects on the visual acuity are mild. These patients can wear colored contact lens to relieve photophobia symptoms. However, the lens opacity may often progress and impact vision, which requires surgery. Due to the congenital structural anomalies and increased complications associated with cataract surgery in this group, careful preoperative planning should be done. Li et al. [13] performed cataract surgery in 12 patients (24 eyes) with congenital aniridia. Most patients had some improvement in visual acuity and quality.

In our study, no lens dislocation occurred. CTR implantation was performed to stabilize the zonules and prevent intraocular lens dislocation in the future. Postoperative visual acuity generally improved.

**Fig. 3:**
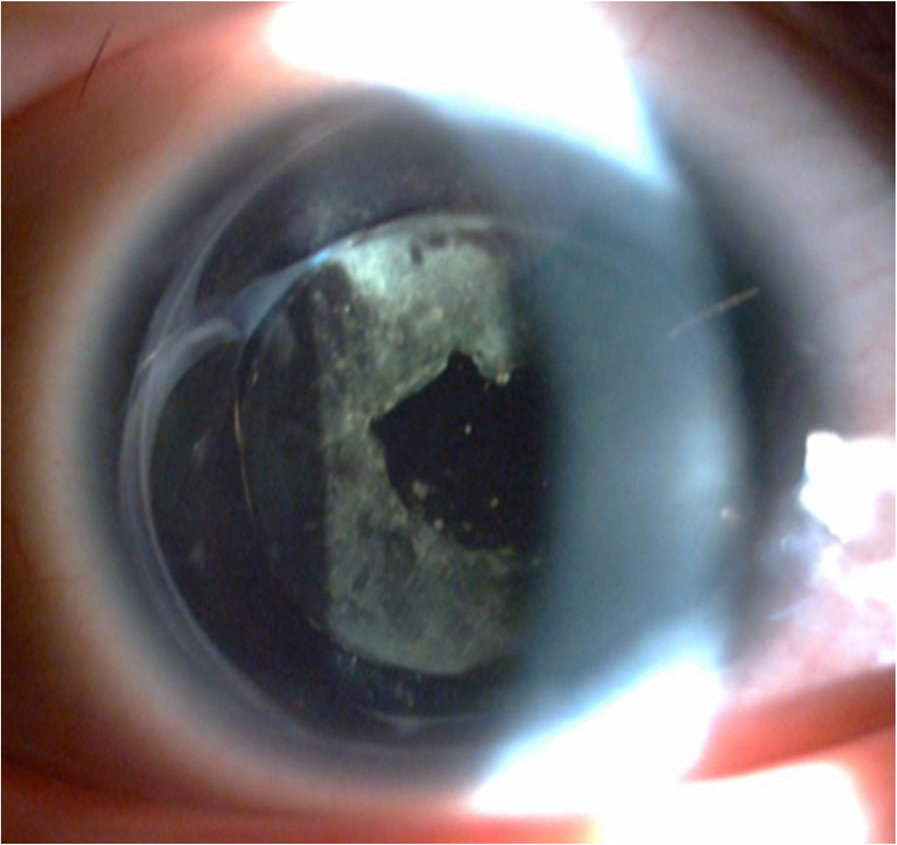
The artificial pupil formed at 12 months after the PCO treated by YAG laser

In hopes of avoiding secondary glaucoma, corneal endothelial decompensation or other complications, as well as to alleviate the postoperative symptoms of photophobia, the CCC diameters in this group of patients were less than 6 mm. The anterior capsule may form an artificial pupil after fibrosis at 12 months after the PCO treated by YAG laser (Fig. 3). Moreover, the light absorbing yellow posterior chamber IOL may exert some light-shielding effects. Wearing cosmetic contact lenses postoperatively can also alleviate some of the photophobic symptoms. There were no instances of secondary glaucoma or corneal endothelial decompensation in our case series.

While iris prosthetic devices have been used to reduce the postoperative photophobic symptoms in patients with congenital aniridia and cataract, it was found to be associated with postoperative secondary glaucoma and corneal endothelial decompensation after long-term follow-up [6, 11, 14–17]. Reinhard et al. [11] reported outcomes of black diaphragm IOL implantation in 19 eyes with congenital aniridia. Deterioration in glaucoma occurred postoperatively in 4 out of 5 eyes with preoperative glaucoma whereas 4 out of 14 eyes without preoperative glaucoma developed postoperative chronic glaucoma. It was postulated that although glaucoma is a common endogenous complication of congenital aniridia [18], the blood–aqueous barrier (BAB) may be altered by the black diaphragm aniridia IOL thereby accelerating glaucoma progression.

There are some surgical tips for cataract surgery in patients with congenital aniridia. Firstly, the CCC must be treated with considerable care. Histological studies have demonstrated that the anterior capsules of patients with congenital aniridia are thin and fragile with degenerative changes in the epithelial cells [19, 20], which may predispose to the occurrence of capsular tears. It has been reported that the use of CTR can significantly reduce the probability of postoperative lens dislocation, PCO and anterior capsule fibrosis [21]. Moreover, patients with congenital aniridia are usually young, so the incidence of PCO is very high. However, many of these patients may have nystagmus that poses difficulty during YAG laser treatment. One possible option is to perform posterior CCC with anterior vitrectomy at the time of cataract surgery in these eyes.

In summary, the visual acuity and quality of vision in patients with congenital aniridia complicated with cataract can be improved through a carefully planned surgery. An appropriate individualized surgical method should be selected to minimize complications and give the best chance of postoperative success. Treatment of congenital aniridia and cataract with phacoemulsification, posterior chamber lens implantation, capsular tension ring placement, and post-operative colored contact lenses can improve the visual acuity and significantly reduce photophobic symptoms.

## Conclusions

Treatment of congenital aniridia and cataract with phacoemulsification, posterior chamber lens implantation, capsular tension ring placement, and post-operative colored contact lenses are effective for some patirents.

## Abbreviations

BAB: The blood–aqueous barrier
BCVA: Best-corrected visual acuity
CCC: Continuous circular capsulorhexis
CTR: Capsular tension ring
IOL: Intraocular len
IOP: Intraocular pressure
OCT: Optical coherence tomography
PCO: Posterior capsule opacification
